# Characteristics of choroidal neovascularization in elderly eyes with high myopia not meeting the pathologic myopia definition

**DOI:** 10.1038/s41598-022-18074-2

**Published:** 2022-08-13

**Authors:** Kaori Sayanagi, Satoko Fujimoto, Chikako Hara, Yoko Fukushima, Ryo Kawasaki, Shigeru Sato, Hirokazu Sakaguchi, Kohji Nishida

**Affiliations:** 1 Department of Ophthalmology E7, Osaka University Graduate School of Medicine, 2-2 Yamadaoka, Suita, 565-0871 Japan; 2 Hawaii Macula and Retina Institute, 98-1079 Moanalua Road, Suite 470, 96701 Honolulu, Hawaii USA; 3 Integrated Frontier Research for Medical Science Division, Institute for Open and Transdisciplinary Research Initiatives (OTRI), Osaka University, Suita, Japan

**Keywords:** Biomarkers, Diseases

## Abstract

The META-Analysis of Pathologic Myopia Study group proposed a new classification system for myopic maculopathy (MM) with pathologic myopia (PM) defined as MM equal to/more serious than diffuse atrophy or the presence of plus lesions and myopic choroidal neovascularization (mCNV) defined as CNV in the eye with PM. However, CNV in elderly eyes with high myopia (HM) not meeting the PM definition (high-myopia CNV) are not classified as age-related macular degeneration (nAMD) or mCNV. This retrospective study included 39 eyes with high-myopia CNV, 20 eyes with mCNV, and 20 eyes with AMD. All patients were at least 40 years old. We compared the clinical characteristics and treatment outcomes among three groups. The high-myopia CNV group had significantly more CNV types, shorter axial length and fewer lacquer cracks (*P* < 0.0001, respectively); larger baseline greatest linear dimension (*P* = 0.0002), more fellow-eye drusen (*P* = 0.0106), more men (*P* = 0.0029), and more treatments (24 months, *P* = 0.0098) compared to the mCNV group. Compared with the nAMD group, the high-myopia CNV group was significantly younger (*P* = 0.0041), and had fewer CNV types (*P* = 0.0316), more lacquer cracks (*P* = 0.0079) and fewer drusen (affected-eye, *P* = 0.0006 and fellow-eye, *P* = 0.0222), and fewer treatments (24 months, *P* = 0.0030). Because the CNV in elderly eyes with HM not meeting the PM definition is classified as combined mCNV and nAMD, the clinical and angiographic findings are critical to determine the treatment strategy.

Pathologic myopia (PM), the leading cause of blindness worldwide especially in East Asian countries^1^, is mainly due to the development of different types of myopic maculopathy (MM). In the Tajimi Study, myopic macular degeneration was the leading cause of blindness in Japanese residents aged 40 years and older^2^. In 2015, the META-Analysis for Pathologic Myopia (META-PM) Study Group proposed a new classification system of MM subdivided into 5 categories: (0) no maculopathy; (1) tessellated fundus; (2) diffuse atrophy; (3) patchy atrophy; and (4) macular atrophy. Three plus lesions included lacquer cracks (LCs) and myopic choroidal neovascularization (mCNV). PM is defined as MM category 2 or higher with the presence of a plus sign or posterior staphyloma^3^. mCNV is defined as CNV that occurs in PM^4–7^. mCNV is a severe vision-threatening complication of PM; Yoshida et al. reported that the visual acuity (VA) deteriorates to 20/200 or worse in about 96% of eyes over 10 years without treatment^8^. The first-line therapy for mCNV is anti-vascular endothelial growth factor (VEGF) therapy. Several clinical trials have reported short-term favorable outcomes after anti-VEGF therapy; however, the long-term outcomes remains challenging^4–6,9–12^.

Age-related macular degeneration (AMD), another leading cause of irreversible visual impairment^13^. AMD is classified as neovascular (wet) (nAMD) and non-neovascular (dry), or a mixture of both^14,15^. Because most AMD-related blindness is associated with nAMD, anti-VEGF agents are currently the preferred option for managing patients with nAMD and set the benchmark for all new treatment options in nAMD. Several pivotal phase III studies have reported unprecedented improvements of one to two lines of vision lasting up to 2 years, and the Fight Retinal Blindness Study Group reported the favorable long-term outcomes of anti-VEGF for nAMD^16–22^.

Although CNV also occurs in elderly eyes with HM not meeting the PM definition, it often is treated as mCNV because many studies have defined mCNV only based on the ocular axial length (AL) or equivalent sphere value. In addition, these eyes have been excluded from AMD clinical studies. Therefore, the clinical features and treatment outcomes have rarely been reported. The current study clarified the clinical characteristics and treatment outcomes of CNV in elderly patients with HM that does not meet the PM definition (high-myopia CNV) compared with mCNV and nAMD.

## Results

**Figure 1:**
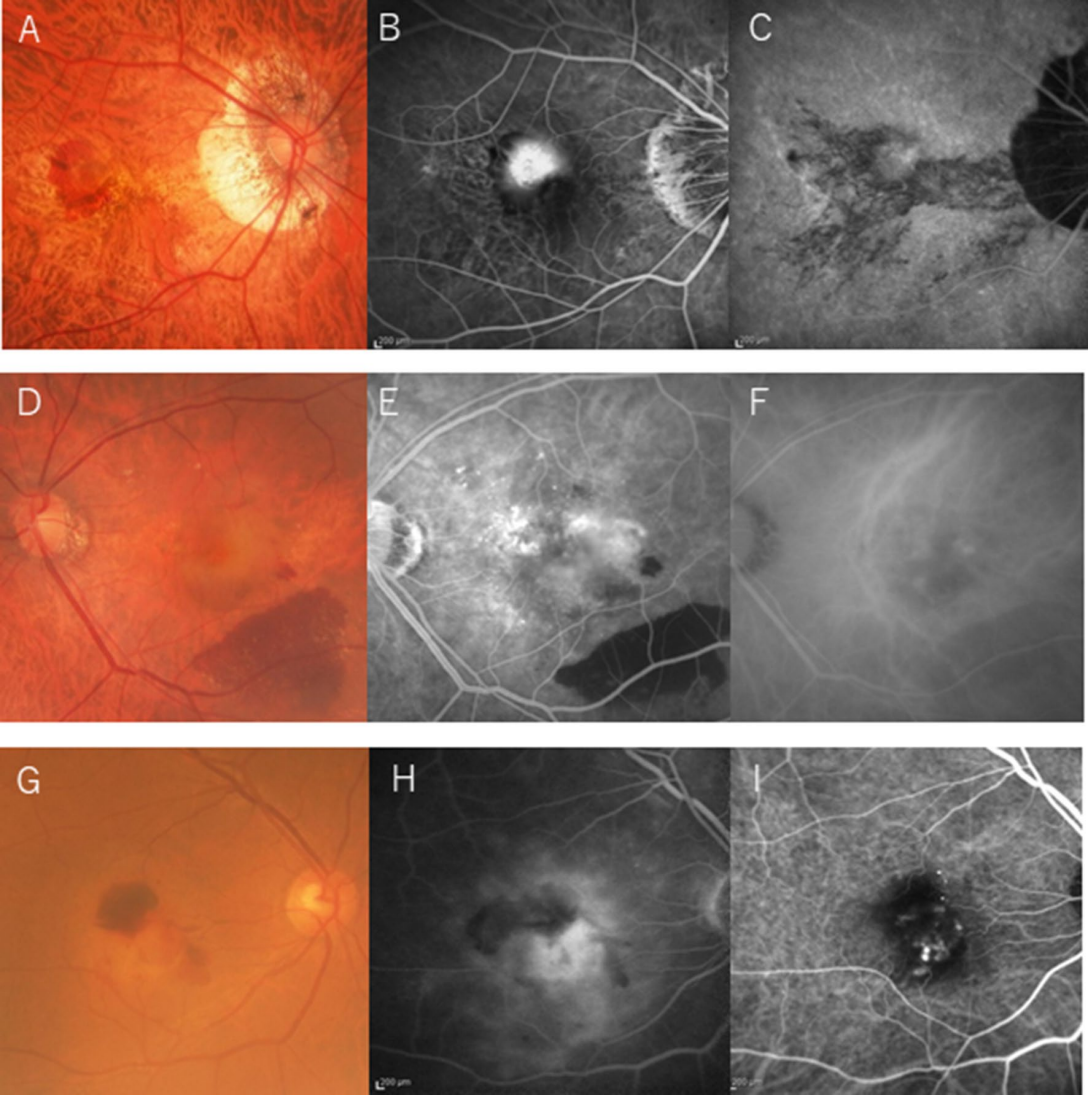
Representative cases of the three groups. (**A**–**C**) The mCNV group has more severe chorioretinal atrophy than diffuse choroidal atrophy (**A**), fluorescein angiography (FA) shows classic CNV (**B**), and ICGA shows LCs (**C**). (**D**–**F**) The high-myopia CNV group shows no chorioretinal atrophy (MM less than category 2) (**D**), FA shows occult CNV and blockage due to subretinal hemorrhage (**E**), and ICGA shows polyp lesions (**F**). (**G**–**I**) The AMD group has an AL of less than 26.5 mm, no chorioretinal atrophy on fundus photography (**G**), occult CNV on FA (**H**), and polyp lesions on ICGA (**I**).

Twenty eyes of 19 patients were diagnosed with high-myopia CNV, 39 eyes of 38 patients with mCNV, and 356 eyes of 355 patients with nAMD. All patients were at least 40 years old. For nAMD, 20 randomly selected eyes of 356 eyes were included in the analysis. The characteristics of the three groups are summarized in Table 1. Representative cases are shown in Fig. 1. The mean follow-up period was 48.1 ± 33.5 months (range 4–130 months), and the initial drugs used were bevacizumab (Avastin, Genentech Inc., South San Francisco, CA, USA) in nine eyes (8 eyes had mCNV and 1 eye had high-myopia CNV), ranibizumab (Lucentis, Genentech Inc.) in 22 eyes (12 eyes had mCNV, 6 eyes had high-myopia CNV and 4 eyes had nAMD) including one eye with high-myopia CNV treated with photodynamic therapy, and aflibercept (Eylea, Regeneron, Tarrytown, NY, USA) in 48 eyes (19 eyes had mCNV, 13 eyes had high-myopia CNV and 16 eyes had nAMD). During the 2 years after the first treatment, one eye of a patient with mCNV developed the multiple evanescent white dot syndrome, one eye of a patient with mCNV underwent pars plana vitrectomy for myopic foveoschisis, 11 eyes underwent cataract surgery (4 eyes had mCNV, 2 eyes had high-myopia CNV and 5 eyes had nAMD); and one eye of the patient with high-myopia CNV underwent gas injection for a massive subretinal hemorrhage.

**Table 1:** Comparison of patient background between high-myopia CNV versus mCNV and versus nAMD.

Characteristics	High-myopia CNV (n = 20)	versus mCNV (n = 39)		versus nAMD (n = 20)	
			*P* value		*P* value
Age (years)	67.2 ± 11.7	68.9 ± 9.5	0.8059	77.4 ± 6.2	**0.0041**
Sex (male/female)	7/12	8/30	**0.0029**	8/12	0.1481
AL (mm)	27.5 ± 1.0	29.0 ± 1.4	** < 0.0001**	22.3 ± 0.63	** < 0.0001**
**CNV type (eyes)**			** < 0.0001**		**0.0316**
Classic	9 (45%)	39 (100%)		2 (10%)	
Occult	5 (25%)	0 (0%)		6 (30%)	
PCV	6 (30%)	0 (0%)		8 (40%)	
RAP	0 (0%)	0 (0%)		4 (20%)	
Baseline GLD (µm)	2,704.2 ± 1653*	1075.6 ± 871.0*	**0.0002**	2886.6 ± 2093	0.9217
Baseline BCVA (logMAR)	0.358 ± 0.29	0.423 ± 0.459	0.7478	0.371 ± 0.383	0.9026
**Presence of drusen (eyes)**					
Affected eye	1 (5%)	0 (0%)	0.3390	11 (55%)	**0.0006**
Fellow eye	4 (20%)	0 (0%)	**0.0106**	11 (55%)	**0.0222**
*Presence of LCs*	6 (30%)	37†(97%)	** < 0.0001**	0 (0%)	**0.0079**
**MM category (eyes)**				NA	
0	6 (30%)	0 (0%)			
1	14 (70%)	0 (0%)			
2	0 (0%)	27 (69%)			
3	0 (0%)	12 (31%)			
4	0 (0%)	0 (0%)			

The values are expressed as the mean ± standard deviation.

Significant values are in bold.

*AL* axial length, *RAP* retinal angiomatous proliferation, *logMAR*, logarithm of the minimum angle of resolution.

*Within the number of cases excluding one eye that did not undergo fluorescein angiography.

^†^Within the number of cases excluding one eye that did not undergo ICGA.

### Clinical characteristics

Compared to the mCNV group, the high-myopia CNV group had significantly more men (*P* = 0.0029), significantly shorter AL (*P* < 0.0001), significantly more CNV types (*P* < 0.0001), significantly greater linear dimension (GLD) (*P* = 0.0002), significantly fewer LCs (*P* < 0.0001), and significantly more drusen in the fellow eyes (*P* = 0.0106). Compared to the nAMD group, the high-myopia CNV group was significantly younger (*P* = 0.0041), and had significantly fewer CNV types (*P* = 0.0316), significantly more LCs (*P* = 0.0079), and significantly fewer drusen in the both affected and fellow eyes (*P* = 0.0006 and *P* = 0.0222, respectively).

Table 2 shows the clinical characteristics of the patients with high-myopia CNV. When the eyes with classic CNV (n = 9) and other CNVs (occult CNV and polypoidal choroidal vasculopathy [PCV]; n = 11) were compared, there were no significant differences in the AL (*P* = 0.3616) or the presence/absence of drusen in both affected and fellow eye (*P* = 1.0000 and *P* = 0.0941, respectively); however, the patients with classic CNV were significantly younger (*P* = 0.0473), had significantly smaller GLD (*P* = 0.0004), significantly more LCs (P = 0.0012), and significantly fewer treatments at 12 and 24 months (*P* = 0.0010 and *P* = 0.0128, respectively) compared to the other CNV eyes.

**Table 2:** Comparison of patient characteristics and number of intravitreous injections between classic CNV and other CNV types in patients with high-myopia CNV.

Characteristics	Classic CNV (n = 9)	Other CNV (n = 11)	*P* value
Age (years)	60.5 ± 12.2	72.1 ± 8.6	**0.0473**
Sex (male/female)	3/5	9/2	0.0739
AL (mm)	27.9 ± 1.1	27.3 ± 0.8	0.3616
Baseline GLD (µm)	1011.4 ± 359.0*	3935.3 ± 1669.4	**0.0004**
Baseline BCVA (logMAR)	0.385 ± 0.359	0.335 ± 0.354	0.8778
Presence of LCs	6 (75%)	0 (0%)	**0.0012**
**Presence of drusen**			
Affected eye	0 (0%)	1 (9%)	1.0000
Fellow eye	0 (0%)	4 (36%)	0.0941
Number of IVIs (12 months)	1.7 ± 1.1 (n = 7)	6.5 ± 2.2 (n = 10)	**0.0010**
Number of IVIs (24 months)	3.0 ± 2.4 (n = 4)	10.1 ± 4.6 (n = 9)	**0.0128**

The values are expressed as the mean ± standard deviation.

Significant values are in bold.

*IVIs* intravitreal injections, *logMAR* logarithm of the minimum angle of resolution.

*Within the number of cases excluding one eye that was not performed with fluorescein angiography.

### Treatment outcome

**Figure 2:**
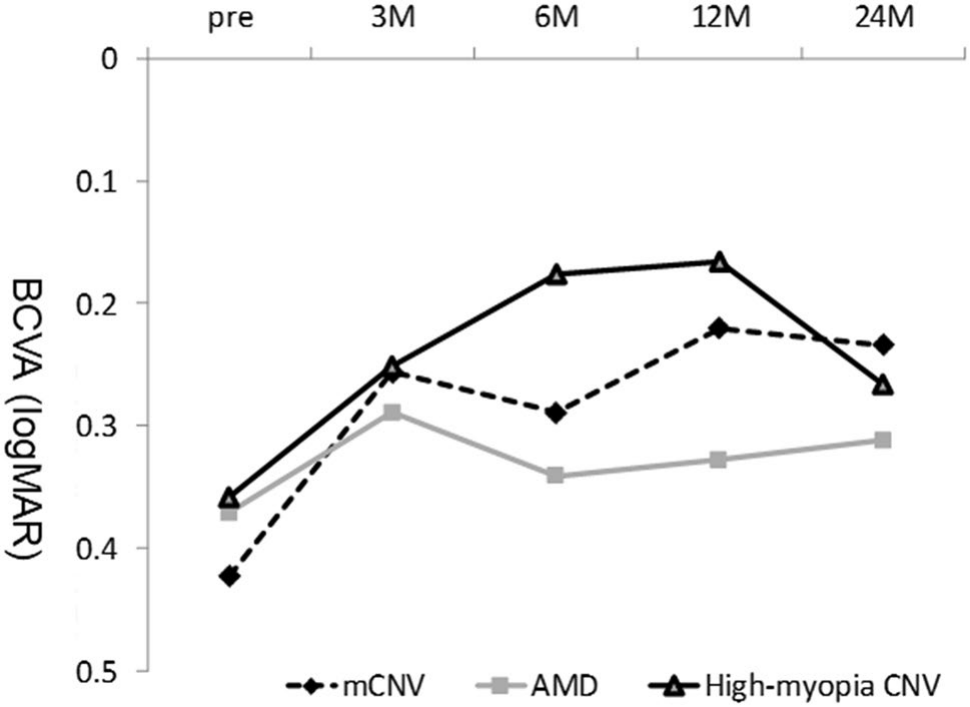
The average VA course of the mCNV, high-myopia CNV, and AMD groups. M, months; logMAR, logarithm of the minimum angle of resolution.

Figure 2 shows the course of the mean best-corrected VA (BCVA) in the three groups after treatment. The high-myopia CNV and nAMD groups had significantly better VA after treatment compared with pre-treatment; however, the difference was no longer significant at 24 months. There was no significant difference between the two groups at any point during the study. In contrast, the VA in nAMD group did not differ significantly from that of pretreatment at any time up to 24 months, and there was no significant difference between the high-myopia CNV and nAMD groups at any time point.

**Figure 3:**
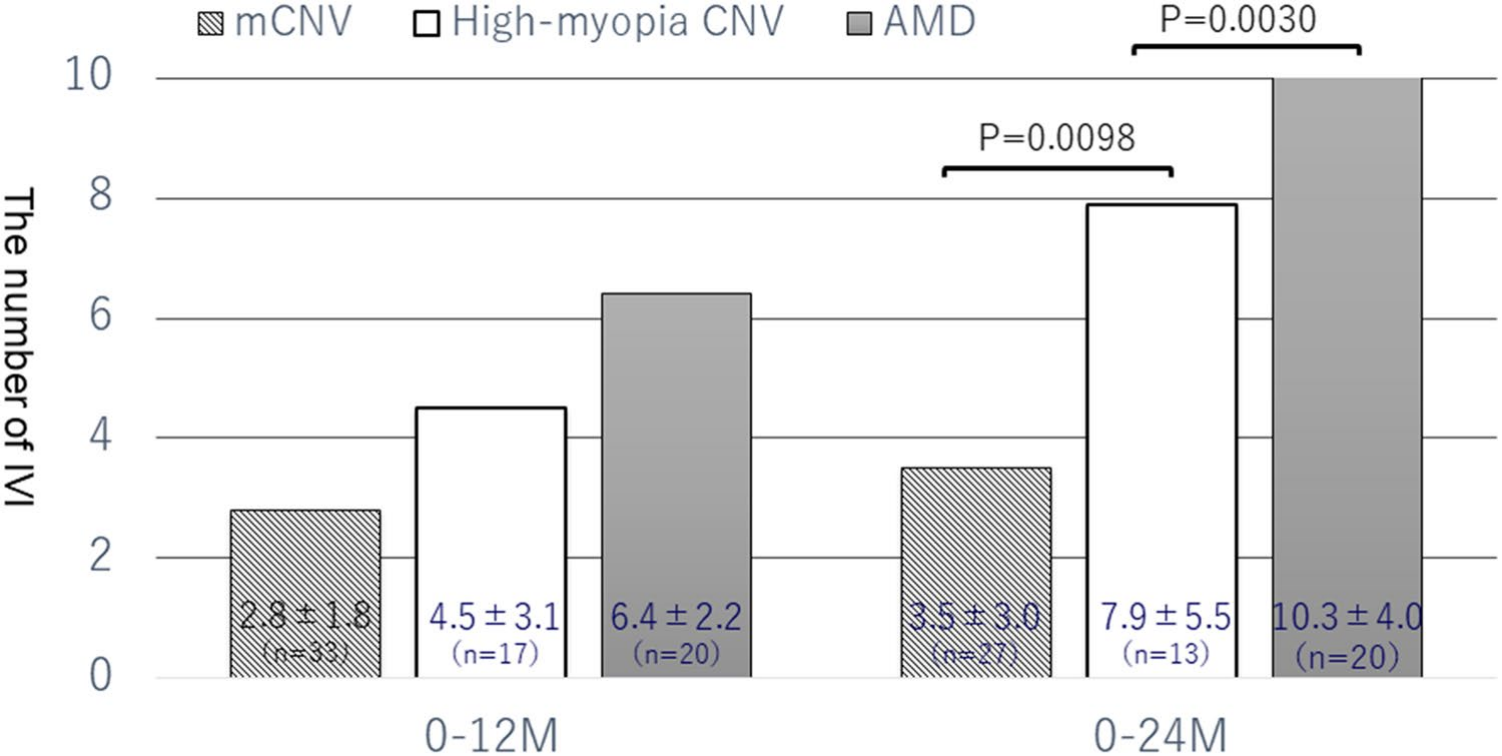
The average number of intravitreal injections in the mCNV, high-myopia CNV, and AMD groups. M, months; IVI, intravitreal injections.

Figure 3 shows the number of treatments at 12 and 24 months in the three groups. A comparison of the high-myopia CNV and mCNV groups showed that former had significantly more treatments than later at 24 months (*P* = 0.0098). Comparisons of the high-myopia CNV and nAMD groups showed that former had significantly fewer treatments than later at 24 months (*P* = 0.0030). In contrast, there is no significant difference in the number of treatments between the high-myopia CNV and mCNV groups at 12 months (*P* = 0.0603) or, and between the high-myopia CNV and nAMD groups at 12 months (*P* = 0.0527).

## Discussion

The current study compared CNV in elderly eyes with HM not meeting the PM definition with mCNV and nAMD to determine the clinical features of high-myopia CNV. Considering the increased frequency of MM in patients older than a minimum of 40 years and the age of onset of AMD, patients older than 40 years were included in this study^23,24^. The results of the comparison with the mCNV group showed that more men were in the high-myopia CNV group, the patients had significantly shorter AL, significantly more occult and PCV, significantly larger GLD, significantly fewer LCs, significantly more fellow-eye drusen, and significantly higher treatment frequency. In addition, compared to nAMD group, significantly younger patients had significantly fewer CNV types, significantly more LCs and significantly less drusen in both the affected and fellow eyes. In other words, it can be inferred that the high-myopia CNV has a mixture of both nAMD and mCNV characteristics. The CNVs that developed in highly myopic eyes of patients younger than 40 years, which were excluded from the present study, may have different characteristics from the elderly patients and require further investigation.

The development of MM is recognized as a major cause of visual impairment worldwide^3,4,25^. However, because a standard classification of MM has not been established, the definitions differ among studies, making it difficult to perform a metanalysis. In 2015, an international panel of researchers in myopia, the META-PM Study Group, proposed a new classification of MM based on color fundus photography that defined PM as MM category 2 or higher with the presence of a plus sign or posterior staphyloma, and defined mCNV, which is characterized as a plus lesion in the new classification, as the presence of CNV in the PM eye^3,4,8^. mCNV is characterized by small classic CNV lesion, usually with minimal exudative changes, and mostly accompanied by LCs on indocyanine green angiography (ICGA) images^4–6^. CNV is rarely accompanied by a pigment epithelial detachment or drusen. Similar to mCNV, nAMD causes CNV in the macula, which is also a cause of severe visual loss worldwide^13^. nAMD is often characterized by the presence of various types of CNV, i.e., occult, classic, polypoidal choroidal vasculopathy, and retinal angiomatous proliferation; various CNV sizes and drusen in the posterior pole^14,15^. The nAMD has exudative changes that are vigorous and present in a variety of locations within the retina and subretina and under the retinal pigment epithelium. The exact pathogeneses of mCNV and nAMD are unknown; however, they are thought to be distinct, i.e. mCNV is thought to result from mechanical damage to the retinal pigment epithelium, Bruch's membrane, and choroid due to ocular axial prolongation as myopia progresses^26–33^, whereas nAMD is thought to result from a complex multifactorial interaction between metabolic, functional, genetic, and environmental factors with age^34,35^. The response to anti-VEGF therapy also differs between mCNV and nAMD. mCNV requires fewer intravitreal injections to suppress CNV activity, whereas nAMD requires multiple intravitreal injections over a longer period of time^4–6,9–12,16–22^. The current results suggested that high-myopia CNV does not correspond to the characteristics of either mCNV or nAMD and is independent of the two.

When cases within high-myopia CNV were compared between classic CNV and other CNVs (occult CNV and PCV), significant differences in some clinical features were seen (Table 2). Compared to the other CNV eyes, the patients with classic CNV were significantly younger; and had a significantly smaller GLD, significantly more LCs, and had received significantly fewer treatments at 12 and 24 months. The classic CNV cases showed mCNV-like features, while the other CNV cases had nAMD-like features. Since there was no difference in AL between the two groups, we speculated that the CNVs of HM not meeting the PM definition may be a mixture of both mCNV and nAMD. In the present study, in the high-myopia CNV group, the number of treatments was significantly higher in patients with occult CNV and PCV than in patients with classic CNV. Therefore, it may be appropriate to follow the treatment strategy for nAMD when occult CNV or PCV is seen on angiography, even if the AL of the eye fulfills the definition of HM.

Another important aspect of the pathogenesis of high-myopia CNV is the progression of MM. Hayashi et al. followed 806 eyes with high myopia for 5 to 32 years and reported the progression pattern of MM in the long term^25^, i.e., 40.6% had progression of MM during a mean follow-up period of 12.7 years. Hence, high-myopia CNV in this report is defined as CNV occurring in HM but less than category 2 MM; however, some cases may progress to category 2 or higher MM during follow-up, and the diagnosis may be divided into mCNV or high-myopia CNV depending on the time of diagnosis in some cases. In addition, although the present study excluded patients younger than 40 years, these patients often have category 1 or 0 MM and are not classified as having mCNV according to the META-PM Study Group criteria^3^. However, some reports have suggested that fundus findings that are suggestive of PM may already be present in early childhood, so the diagnosis of mCNV in patients younger than 40 years may require further investigation^36^.

Corbelli et al. investigated the incidence and characteristics of AMD in 874 of 442 patients with HM and reported that 11.9% had AMD, half of which were cases of dry AMD and the other half nAMD^37^. They also reported that 75% of the nAMD cases had type 1 CNV, and the mean number of intravitreal injections during the first year in treatment-naive eyes was 3.8 ± 1.5. Our study was similar to that report in that there were more PCV and occult CNV (type 1 CNV) cases than classic CNV (type 2 CNV) cases in the high-myopia CNV group, and more drusen were seen in the PCV and occult CNV cases. However, the mean number of intravitreal injections during the first year in the high-myopia CNV group was 4.5 ± 3.1 and in the high-myopia CNV group 1.7 ± 1.1 for classic CNV and 6.5 ± 2.2 for other CNVs. The numbers of treatments for PCV and occult CNV were higher than those reported by Corbelli et. al. The reason for the discrepancy between our results and theirs may be that their inclusion criteria included an ocular AL exceeding 25.5 mm or equivalent sphere value below − 6 diopters (D), which differed from our inclusion criteria that focused on MM progression. The number of treatments may have been affected by the difference in administrative methods, i.e., they used pro re nata and we used a modified treat-and-extend (TAE) regimen.

The current study had several limitations including its retrospective design and a small sample size. The difference in the anti-VEGF drugs and additional treatment regimens may have affected the results. Future prospective longitudinal studies with more patients during long follow-up periods are needed to confirm the current results. A small number of patients underwent photodynamic therapy or surgery during the study course, which may have affected the number of intravitreal injections and visual outcomes.

In summary, we clarified the clinical characteristics and outcomes of CNV in elderly patients with HM not meeting the PM definition (high-myopia CNV) by comparing them with mCNV and nAMD and showed that high-myopia CNV may be a combination of mCNV and nAMD. To the best of our knowledge, this is the first report on elderly patients with CNV occurring in HM not meeting the PM definition. Since the courses and outcomes of treatment for high-myopia CNV differ depending on the CNV type, we should not simply diagnose mCNV, even in HM, but should perform angiography and develop a treatment strategy.

## Methods

This was a retrospective, observational study based on the medical records of patients treated at Osaka University Hospital Osaka, Japan. The patients were classified into the following three groups: (1) the mCNV group, in which CNV occurred in eyes with AL of 26.5 mm or greater and met the definition of PM of category 2 or greater in MM. (2) the high-myopia CNV group, in which CNV developed in eyes with AL of 26.5 mm or greater and did not meet the definition of PM, that means category 0 or 1 in MM. (3) the AMD group, in which CNV occurred in eyes with AL of less than 26.5 mm and there was no myopic changes in the fundus. This study included 20 eyes of 19 cases in high-myopia CNV group, 39 eyes of 38 cases in mCNV group, and 20 eyes of 20 cases in AMD group. All patients included in the analysis were at least 40 years old, had not been treated previously for CNV, and were treated with intravitreal therapy starting after January 2010 with at least 4 months of follow-up since starting treatment. The definition of HM was a refractive error of − 6.0 D or higher or an AL of 26.5 mm or longer, and two specialists (K.S. and S.F.) categorized the MM according to the classification proposed by the META-PM Study Group^3^. For the nAMD group, 20 eyes were selected randomly from 356 eyes that met the inclusion criteria. The exclusion criteria included tilted macular syndrome; ocular turbidity such as severe cataract, severe vitreous opacity, and/or severe hemorrhage that caused the fundus and angiography images to be blurry; and a history of pars plana vitrectomy. Eligible and noneligible cases were determined based on fundus photographs, optical coherence tomography (OCT) findings, and angiographic findings. At each follow-up visit, the patients underwent a complete ophthalmic examination, which included an assessment of the BCVA, slit-lamp biomicroscopy, dilated funduscopy, color fundus photography (TRC-50DX, Topcon Corporation, Tokyo, Japan), and OCT (swept-source OCT DRI OCT-1 Atlantis, Topcon Corporation). The decimal BCVA was measured using the Landolt chart and was expressed as the logarithm of the minimal angle of resolution. Angiography was performed with both fluorescein angiography and ICGA at baseline using the Heidelberg Retina Angiograph + OCT (Heidelberg, Germany) and a fundus camera (TRC-50DX, Topcon Corporation), and the AL was measured with the Zeiss IOLMaster (Carl Zeiss Meditec, Oberkochen, Germany).

The research adhered to the tenets of the Declaration of Helsinki. The institutional review board of Osaka University Hospital approved this retrospective study. Informed consent was obtained in the form of an opt-out option on the website.

### Anti-VEGF treatment

After topical anesthesia was applied, an injection of an anti-VEGF drug, aflibercept, or ranibizumab and/or bevacizumab was administered 3.5 to 4.0 mm posterior to the corneal limbus into the vitreous cavity using a 30-gauge needle. Prophylactic topical antibiotics were applied for 3 days after the injection. After the initial treatment, additional treatment in the mCNV groups was administered as needed (PRN), and in the AMD group it was based on a modified TAE regimen, which has been reported previously^38^. The additional treatment in the high-myopia CNV group was administered by either PRN or TAE based on the decision of the responsible physician. Briefly, the patients were followed monthly after the induction dose and the TAE regimen began when the need for additional treatment was diagnosed. The criteria for additional treatment were determined based on objective/subjective visual declines or new hemorrhage or recurrent exudative changes seen on OCT. The institutional review board of Osaka University Hospital approved the use of bevacizumab.

### Statistical analysis

Statistical analysis was performed using JMP Pro software version 14.1.0 (SAS Institute, Cary, NC, USA). Fisher’s exact test was used to analyze the non-parametric data. Pearson’s chi-square test was used to analyze the proportion of CNV types and Wilcoxon t-test for nonparametric numerical data. *P* < 0.05 was considered significant.

## Data Availability

All data generated or analyzed during this study are included in this published article.
